# Antimalarial Activity of *Meriandra dianthera* Leaf Extracts in *Plasmodium berghei*-Infected Mice

**DOI:** 10.1155/2020/8980212

**Published:** 2020-02-12

**Authors:** Kalay Hagazy, Gereziher G. Sibhat, Aman Karim, Gebretsadkan H. Tekulu, Gomathi Periasamy, Mebrahtom G. Hiben

**Affiliations:** ^1^Department of Pharmacy, College of Health Sciences, P.O. Box 298, Aksum University, Axum, Ethiopia; ^2^Department of Pharmacognosy, School of Pharmacy, College of Health Sciences, Mekelle University, P.O. Box 1871, Mekelle, Ethiopia

## Abstract

**Objective:**

To evaluate the antimalarial effect of aqueous methanolic extract and solvent fractions of *Meriandra dianthera* leaves against *Plasmodium berghei* in mice model.

**Method:**

*M. dianthera* leaves were extracted with 80% methanol and dried. The dried crude extract was then defatted and further fractionated with chloroform, ethyl acetate, and butanol. Acute oral toxicity test was performed as per the Organization for Economic Cooperation and Development guideline 425. Peter's 4-day suppressive test was used to determine the *in vivo* antimalarial activity of the extract and fractions.

**Result:**

The crude leaf extract of *Meriandra dianthera* leaves against *P* < 0.001) chemosuppression compared to the negative control. Both the extract and fractions were able to prevent *P. berghei* induced body weight loss and body temperature reduction and also increased the survival time of the mice as compared to the negative control. The aqueous methanolic leaf extract of *M. dianthera* leaves were extracted with 80% methanol and dried. The dried crude extract was then defatted and further fractionated with chloroform, ethyl acetate, and butanol. Acute oral toxicity test was performed as per the Organization for Economic Cooperation and Development guideline 425. Peter's 4-day suppressive test was used to determine the

**Conclusion:**

The extracts of *M. dianthera* leaves showed promising antimalarial activity, with no sign of toxicity and therefore may support its traditional use for the treatment of malaria.*M. dianthera* leaves were extracted with 80% methanol and dried. The dried crude extract was then defatted and further fractionated with chloroform, ethyl acetate, and butanol. Acute oral toxicity test was performed as per the Organization for Economic Cooperation and Development guideline 425. Peter's 4-day suppressive test was used to determine the

## 1. Introduction

Malaria is a preventable and curable disease, yet it remains an overwhelming tropical disease, with high infection and mortality data [1]. It is caused by a protozoan parasite belonging to the genus *Plasmodium* [2]. More than one hundred different species of *Plasmodium* exist and produce malaria in many types of animals. Five of the *Plasmodium* species can cause malaria in humans (*Plasmodium falciparum*, *P. vivax*, *P. ovale*, *P. malariae*, and *P. knowlesi*) and two of these, *P. falciparum* and *P. vivax*, were reported as the greatest threat and most frequent human malaria causing species [3]. According to the World Health Organization (WHO) Malaria Report 2019, roughly 228 million cases and 405,000 deaths from malaria were estimated in the year 2018 worldwide. More than 90% of the cases and deaths were in Africa and approximately 70% of the global deaths from malaria were in under-five children [4].

Despite using insecticide-treated bed nets, artemisinin-based combination treatments, and indoor residual spraying interventions, malaria accounts for 17% of outpatient visits, 15% of hospital admissions, and 29% of in-patient deaths in Ethiopia [5]. The Federal Ministry of Health (FMOH) of Ethiopia estimates that there are about 12 million suspected malaria cases annually. *P. falciparum* and *P. vivax* account roughly for 65% and 35% of malaria cases, respectively [6]. About 68% (57.3 million) of the country's population is at high risk of being infected with malaria.

Even though the WHO has planned to eradicate malaria, it continues to be the top leading health problems in Africa. This is attributed to the emergence and spread of drug-resistant parasites, insecticide-resistant *Anopheles*, absence of a successful malaria vaccine, a geosociopolitic ruckus that increases travel [7], and rapidly raising distribution of counterfeit antimalarial drugs [8].

In malaria-endemic countries, natural and traditional medicines are commonly used to treat malaria. Over 160 families of plants with over 1200 species are recognized as traditional medicines used for malaria treatment. Of these, several medicinal plants have been scientifically confirmed by *in vitro* and/or *in vivo* tests for the claimed activity against malaria [9]. *Meriandra dianthera* (Roth.) (syn. *M. benghalensis*, Labiatae), locally known as “mesaguh” (Tigrigna) [10], is a branched aromatic shrub which grows up to 2 m in height. It is native to the high plateau of Ethiopia, Eritrea, Yemen, and Saudi Arabia. The leaf extract of *M. dianthera* is widely used as a folk medicine for the treatment of malaria, diabetes, diarrhea, ascariasis, and hypertension [10, 11]. The aqueous leaf extract of the plant in the form of juice is taken for five days to treat malaria in Seharti Samre, Ethiopia [11].

Limited scientific works exist that intend to verify the traditional claims of this plant. Four abietane diterpenoids isolated from *M. dianthera* root extract were reported to have a potent cytotoxic effect against different cancer cell lines as well as antibacterial activities [12]. Moreover, an essential oil extracted from *M. dianthera* aerial parts was reported to demonstrate promising anticancer effect against all cancer cell lines (IC_50_ values ranged from 83.6 to 91.2 *μ*g/mL) and considerable antibacterial, antifungal, and antioxidant properties [13]. Furthermore, methanolic leaves extract of *M. dianthera* showed significant *in vitro α*-amylase and *α*-glucosidase inhibitory activities as well as *in vivo* antidiabetic effect in alloxan-induced diabetic rats [14, 15]. As there is no scientific work that verifies the traditional antimalarial claim of *M. dianthera*, the present study was aimed to investigate the *in vivo* antimalarial activity of *M. dianthera* leaf extract using mice model.

## 2. Materials and Methods

### 2.1. Plant Material Collection

The leaves of *M. dianthera* were collected in November 2016 from Maynebri, which is located around Hiwane town in Tigray (this town has a latitude and longitude of 13°6′18″N and 39°29′48″E), Ethiopia. The plant was authenticated by Mr. Melaku Wondafrash and a voucher specimen (Collection No. KH001) was deposited in the National Herbarium, Addis Ababa University. The leaves were shade dried at room temperature and powdered with mortar and pestle.

### 2.2. Experimental Animals

Swiss albino female mice 8–12 weeks old were used to perform the acute oral toxicity test. Male Swiss albino mice (weighing 24–30 g and aged 6–8 weeks) were used in the antimalarial activity study. The mice were housed in a ventilated room, allowed to acclimatize for one week before the study and exposed to a 12 h light/dark cycle and had free access to standard pellets and water *ad libitum*. Chloroquine-sensitive *Plasmodium berghei* ANKA strain (provided by Ethiopian Public Health Institute) was used for the experiment and maintained by subsequent passage of blood from infected donor mouse to the healthy mouse every 5 days.

### 2.3. Preparation and Fractionation of the Crude Extract

Powdered plant material (1.3 kg) was soaked in 6.5 L of 80% methanol (Carlo ERBA reagents SAS, France) and occasionally kept on an orbital shaker at 130 rpm for three days. After 72 h, the extract was filtered and the residue was macerated twice in a similar manner. The filtrates were combined, concentrated with a rotary evaporator (Stuart, SO1, UK), and dried in an oven (Genlab, England) at 40°C.

The crude extract was fractionated according to Kupchan's method by suspending 25 g of the crude extract in aqueous methanol (90%) and subsequently defatted with petroleum ether (Blulux Laboratories Ltd., India) and dried. Further, 22 g of dried defatted extract was suspended in distilled water (200 ml) and fractionated with chloroform (Carlo ERBA reagents SAS, France), ethyl acetate (Carlo ERBA reagents SAS, France), and *n*-butanol (Carlo ERBA reagents SAS, France) four times for each solvent (200 ml) [16, 17]. All four fractions were concentrated using a rotary evaporator, dried in an oven at 40°C, and stored in a refrigerator at −4°C in airtight containers.

### 2.4. Acute Oral Toxicity Test

Acute oral toxicity test for the crude extract was conducted on a group of five mice (nulliparous female Swiss albino) following the Organization for Economic Cooperation and Development (OECD) 425 guidelines to determine the safe dose for the antimalarial activity study [18]. The mice were deprived of food but not water for 3 h before oral administration of a single dose of the crude extract. Initially, one mouse was given the extract orally at a dose of 2000 mg/kg dissolved using 2% tween 80 in distilled water (v/v) by oral gavage and observed for 24 h. Later, the remaining four mice were given the same dose of the extract as the first mouse and were observed continuously for the first 30 min, intermittently for 4 h over a period of 24 h, and daily for 14 days. Mice were observed for change of general behavior and physiological signs of toxicity [18]. Oral acute toxicity study of the solvent fractions was conducted as per the method described for the crude extract.

### 2.5. Experimental Design

A total of 15 groups each containing six mice were used to evaluate antimalarial activity of the crude extract and solvent fractions. The first five groups served for crude extract and the remaining ten groups for the solvent fractions.

### 2.6. In Vivo Antimalarial Activity Test

To evaluate the *in vivo* antimalarial activity of the plant extracts, we adopted Peters' 4-day suppressive test using *P. berghei*-infected mice model [19]. Blood was obtained from the donor mice (that have 20–30% parasitaemia level) through jugular vein puncture and collected in Petri dish containing 0.5% trisodium citrate. The blood was then diluted with physiological saline (0.9%) based on parasitaemia level of the donor mice to prepare a blood suspension in which there were approximately 5 × 10^7^ infected erythrocytes per milliliter. Each mouse was injected intraperitoneally with 0.2 ml of blood suspension.

Three hours after the inoculation of the parasite, the mice in the three treatment groups orally received different doses of the crude extract (200, 400, and 600 mg/kg) for four consecutive days. The negative and positive control groups received vehicle (2% tween 80 in distilled water (v/v)) and 25 mg/kg chloroquine phosphate (EPHARM, Ethiopia), respectively. The fractions, chloroform, ethyl acetate, butanol, and aqueous, were also evaluated for their antimalarial activity by the same procedure at the dose of 200 mg/kg and 400 mg/kg.

Blood was collected from the tail of each mouse on day four, and thin blood smears were prepared on the frosted microscopic slides (Narang Medical Ltd., UK). The smears were fixed with methanol and stained with a 10% giemsa solution, wetted at the top with a drop of oil immersion, and examined under a compound microscope (Optica Microscopes, Italy) with an objective lens of 100x magnification power. The parasitaemia was determined by counting a minimum of four fields per slide with 200 RBC per field approximately [20]. Percent parasitaemia and percent parasitaemia suppression were calculated using the modified formula [21]:

(1) $$\begin{array}{c} \%\text{ parasitaemia }=\;\frac{\text{number of parasitized RBC }}{\text{total number of RBC count }}\;\times \;100,\\ \%\text{ suppression }=\;\frac{\text{parasitaemia level of negative control}-\text{parasitaemia level of test group }}{\text{parasitaemia level of negative control}}\times 100. \end{array}$$

#### 2.6.1. Determination of Body Weight and Temperature

The body weight of each mouse in all groups was recorded on day 0 and after infection on day 4. The rectal temperature of mice was measured with a digital thermometer to see the effect of the extract and fractions on the prevention of body temperature reduction due to malaria [22].

#### 2.6.2. Determination of Mean Survival Time

The mice were monitored daily to see if there is any mortality and the number of days from the time of inoculation of the parasite up to death was recorded for each mouse in all groups throughout the follow-up period. The mean survival time (MST) for each group was calculated as follows [23]:

(2) $$\begin{array}{c} \text{MST }=\frac{\text{sum of the survival time of mice in a group }\left(\text{days}\right)}{\text{total number of mice in that group}}. \end{array}$$

### 2.7. Phytochemical Screening

The crude plant extract was screened to identify various classes of chemical constituents following the methods described by Trease and Evans [24].

### 2.8. Statistical Analysis

The data were analyzed using SPSS version 21. The results are reported as mean ± SEM. One-way analysis of variance (ANOVA) followed by Tukey's HSD *post hoc* test was used to compare results among and within the groups. The results were considered significant when *P* < 0.05.

## 3. Results

### 3.1. Acute Oral Toxicity Test

The crude leaves extract of *M. dianthera* showed no signs of toxicity in mice during 14-day observation period at a dose of 2000 mg/kg, signifying that the oral LD_50_ of the extract is beyond 2000 mg/kg.

### 3.2. *In vivo* Antimalarial Activity of the Crude Extract

The hydromethanolic leaf extract of *M. dianthera* revealed dose-dependent suppressive activity but did not completely clear the parasite on day four (Table 1). Interestingly, analysis of parasitaemia level on day four revealed significant parasite suppression at the doses of 400 mg/kg (*P* < 0.01) and 600 mg/kg (*P* < 0.001) when compared to the negative control group. However, at the dose of 200 mg/kg, no significant chemosuppression was observed. The mice in the positive control group treated with chloroquine phosphate (CQ) at a dose of 25 mg/kg were free of any parasitaemia on day four. 

For survival analysis, no mouse died in the positive control group up to 30^th^ day after treatment, whereas all mice died in the negative control group on the 7^th^ day after infection. The crude extract prolonged mean survival time of the study mice in a dose-dependent manner (Table 1), indicating that the extract might suppress *P. berghei* and hence diminished the overall pathologic effect of the parasite on the study mice. A significantly prolonged survival time compared to the negative control group was observed for groups that received 400 mg/kg/day (*P* < 0.01) and 600 mg/kg/day (*P* < 0.001) of the crude extract except the group treated with 200 mg/kg/day.

Compared to the negative control, the treatment groups received crude extract at all three dose levels which prevented rectal temperature reduction, although statistically not significant. The CQ treated group (25 mg/kg) showed significant (*P* < 0.05) increase in body temperature as compared to the negative control (Table 2).

The crude extract of *M. dianthera* also prevented weight loss in *P. berghei*-infected mice; though statistically not significant, each treatment group showed mean weight increase on the fifth day of infection when compared to the negative control and CQ treated groups (Table 2). But the increase in body weight is irregular that the highest weight gain was seen at the middle dose (400 mg/kg) followed by the high dose (600 mg/kg) and then the low dose (200 mg/kg). This could be due to the presence of appetite suppressant molecule(s) in the crude extract which could not have significant appetite suppressive effect at the lower dose but may suppress the appetite of the animals substantially while increasing the dose of the crude extract as the amount of the molecule(s) increases with dose.

### 3.3. *In vivo* Antimalarial Activity of the Solvent Fractions

All the fractions reduced parasite load as compared to that of the negative control group as observed on day four after treatment. However, at the dose of 400 mg/kg of the chloroform fraction (CF) and aqueous fraction (AF), a statistically significant (*P* < 0.05) reduction in mean parasitaemia level was shown (Table 3). Even though insignificant (*P* > 0.05), an oral dose of the butanol (BF) and ethyl acetate fractions (EF) was also able to reduce the parasitaemia level. The standard drug chloroquine cleared parasitaemia to an undetectable level on the fifth day and the suppressive effect was statistically significant (*P* < 0.001) as compared to the negative control and all dose levels of the four fractions.

All dose levels of the *M. dianthera* solvent fractions moderately prolonged the mean survival time but chloroform fraction at a dose of 400 mg/kg was able to significantly (*P* < 0.01) improve mean survival time of the mice as compared to the negative control group. CF at a dose of 400 mg/kg significantly prolonged survival time (*P* < 0.05) of mice as compared to BF treated and negative control groups. Chloroquine treated mice survived all of the 30-day follow-up, and improvement in survival periods was statistically significant (*P* < 0.001) in relation to the control and all fractions treated groups (Table 3).

As shown in Table 4, a clear difference was observed among infected mice treated with different doses of the four fractions and negative control in preventing the rectal temperature reduction. Among the four fractions, only chloroform fraction (400 mg/kg/) was able to show a statistically significant difference (*P* < 0.01) in preventing the decrease in rectal temperature caused by *P. berghei* infection in mice compared with the negative control. Contrary to this, among the four fractions, EF200 mg/kg showed enhanced rectal temperature reduction significantly (*P* < 0.001) when compared with the AF400 mg/kg and QC25 mg/kg. Despite the difference among groups, dose-dependent protection of rectal temperature reduction was seen in all fractions except in the butanol fraction treated mice.

Further, the analysis of percent change in body weight, between days 0 and 4, revealed significantly greater protection against parasite-induced bodyweight reduction when compared to the negative control (Table 4). All the fractions except that of chloroform showed significant body weight increment as compared with CQ (25 mg/kg) treated group.

### 3.4. Phytochemical Analysis

Preliminary phytochemical screening of the crude extract of *M. dianthera* revealed the presence of alkaloids, saponins, steroids, phenols, tannins, flavonoids, and terpenoids.

## 4. Discussion

In this study, we evaluated the *in vivo* antimalarial activity, safety profile, and chemical constituents of the crude leaf extract and solvent fractions to validate the traditional claim of *M. dianthera* for its use in the treatment of malaria.

In the *in vivo* antimalarial study, we found a dose-dependent chemosuppressive effect by crude leaf extract and solvent fractions of *M. dianthera* against *Plasmodium berghei* compared to the negative control. More significant suppressive effects were noted at 400 mg/kg and 600 mg/kg dose level. The chemosuppression activity of the crude extract was in a dose-dependent manner which could be attributed to the concentration of schizonticidal compounds in the extract [1, 25]. This antimalarial activity observed in *M. dianthera* plant extract is in agreement with the other studies on different plant species of Lamiaceae family. The extract of *Solenostemon monostachyus* progressively reduced parasitaemia induced by chloroquine-sensitive *P. berghei* infection in prophylactic (28.48–71.72%), suppressive (12.52–72.47%), and curative (22.4–82.34%) models in mice [26]. The isolated compound from the ethanol extract of the dried root barks of *Ocimum sanctum* exhibited comparable activity to chloroquine and amodiaquine [27]. From the study of Kirmizibekmez et al., two flavonoid glycosides isolated as the major antimalarial constituents from *Phlomis brunneogaleata* showed activity with IC_50_ values of 5.4 and 12.7 *µ*M against the *Plasmodium falciparum* K1 clones [28].

The crude extract has the highest antimalarial activity from all test samples in this study with chemosupression of 42.28% and 45% (*P* < 0.001) at doses of 400 mg/kg and 600 mg/kg, respectively, compared to the negative control. From the solvent fractions, the aqueous and chloroform fractions (400 mg/kg) exhibited the highest chemosupression (34% and 35.4%) (*P* < 0.05). The ethyl acetate and butanol fractions moderately inhibited the parasite growth but the suppression was not statistically significant compared to the negative control.

Furthermore, the mice treated with crude extract at middle and higher doses as well as CF at a dose of 400 mg/kg survived significantly (*P* < 0.05) as compared to the negative control. In addition, as weight loss and body temperature reduction are the general features of malaria-infected mice [29], the antimalarial active agents are expected to prevent body weight loss in *P. berghei*-infected mice. In this study, the crude leaf extract and solvent fractions of *M. dianthera* prevented weight loss in a dose-dependent manner. Interestingly, all the fractions at the highest dose significantly prevented weight loss which could be due to appetite enhancing and immunomodulatory components in the fractions [30].

Fever is one key manifestation of human malaria but in the case of *P. berghei*-infected mouse model, malaria is associated with a decrease in body temperature [31]. In this experiment, the crude extract and fractions were unable to significantly prevent rectal temperature reduction compared to negative control but the chloroform fraction at 400 mg/kg (*P* < 0.01) significantly prevented temperature reduction compared to crude extract and other fractions.

*In vivo* tests have advantages over *in vitro* tests as it takes into account the possible effects of the test samples as a prodrug and their role in modifying the immune system for the suppression of infection in a living host [1, 32]. Multiple other *in vivo* studies on rodent malaria using diverse plant species from Ethiopia and researchers from other countries have reported antimalarial activity of medicinal plants including*Withania somnifera* [22], *Dodonaea angustifolia* [33], and *Osyris quadripartita* [34]. The scarcity of previous reports pertaining to the antimalarial activity of *M. dianthera* including the relative composition and predominance of its leaf chemicals could not permit a discussion from a comparative perspective. This is the first scientific report that shows the antimalarial activity of the genus *Meriandra*. From the Lamiaceae family, *in vitro* antiplasmodial activity of the seventeen solvent extracts and eleven essential oils of *Salvia* species displayed antiplasmodial activity [35], which is in agreement with this study. Similarly, solvent fractions of *Solenostemon monostachyus*, isolated compounds obtained from *Ocimum sanctum*, and *Phlomis brunneogaleata* have exerted good antiplasmodial activity which are consistent with the results of the current study [26–28]. In the acute toxicity study, after administration of crude leaf extract *M. dianthera*, all the mice were found physically active and no gross behavioral changes or other signs of toxicity were observed till the end of the follow-up period. This indicates that the test extract and solvent fractions may be safe up to a dose of 2000 mg/kg body weight [18].

The preliminary phytochemical tests of crude leaf extract of *M. dianthera* revealed the presence of alkaloids, saponins, terpenoids, tannins, flavonoids, and steroids which were also detected by Demoz and his team in an earlier study [36]. However, further work to isolate active constituents was not performed since the parasite suppression effect by the crude extract was more potent than the different fractions tested which may imply that considering additive and/or synergistic effect may be more important than considering individual components. Yet, previous studies reported essential oils and components therein including camphor, terpineol, pinocarveol, verbenol, caryophyllene oxide, ledol, isolongifolene, borneol, spathulenol, thymoquinone, *β*-eudesmol, and thymol [13] as well as bioactive fatty acid methyl esters including palmitic acid methyl ester, *α*-linolenic acid methyl ester, and other benzene derivatives in *M. dianthera* leaves [14]. These essential oils [37–39] and fatty acid esters were reported to demonstrate significant *in vitro* and *in vivo* antiplasmodial activities [40–42]. Besides the antiplasmodial effect, both the essential oils and fatty acid methyl esters were reported to exhibit *in vitro α*-glucosidase and *α*-amylase inhibitory effects [13, 15]. Also, the essential oils exerted anticancer, antimicrobial, and antioxidant property [13], and the fatty acids exhibited *in vivo* antidiabetic activity in rodent models [14].

Phytochemicals exert their antiplasmodial effect through different mechanisms. For example, alkaloids intercalate with the parasite DNA [43] and flavonoids and phenolics act by various mechanisms which include inhibiting the fatty acid biosynthesis (FAS II) of the parasite [34], elevating the red blood cell oxidation, inhibiting the parasite's protein synthesis, and counteracting the oxidative stress induced by the malaria parasite [44]. The extract and fractions of *M. dianthera* may have exerted their action through the mechanisms mentioned above or by some other unknown mechanism. Hence, the extract and fractions which showed main antiplasmodial activity could be due to additive and/or synergistic action.

## 5. Conclusion

In this study, the crude extract and solvent fractions of *M*. *dianthera* leaves inhibited the growth of *P. berghei* in a dose-dependent manner. The chloroform and aqueous fractions showed the highest chemosuppressive effect among all the extracts. In terms of toxicity, the extracts can be considered safe until the dose of 2000 mg/kg. In addition, this study for the first time scientifically validates the traditional claim of the plant for its antimalarial property. Further phytochemical studies on the chloroform and aqueous fraction are recommended to identify the active constituents, which may contain potential candidates to be developed as an antimalarial drug.

## Figures and Tables

**Table 1:** Effect of the hydromethanolic leaf extract of *M. dianthera* on mean parasitaemia level and mean survival time of *P. berghei*-infected mice.

Test sample	Dose (mg/kg)	% of parasitaemia	% of suppression	MST (days)
NC	10 ml/kg	44.10 ± 1.82	0.00	7.00 ± 00
Extract	200	38.43 ± 3.25^c^^*∗∗*^	14	7.83 ± 0.31
Extract	400	25.77 ± 2.41^a^^*∗*^^c^^*∗∗*^	42.28	8.33 ± 0.33^a^^*∗*^
Extract	600	23.02 ± 1.88^abc^^*∗∗*^	45.52	8.67 ± 0.33^a^^*∗∗*^
CQ	25	00 ± 00^a^^*∗∗*^	100	ND

Results are presented as mean ± SEM; *n* = 6; ^a^when compared to negative control (NC); ^b^when compared to 200 mg/kg of the extract; ^c^when compared to CQ treated group; ^*∗*^*P* < 0.01; ^*∗∗*^*P* < 0.001; CQ = chloroquine phosphate; ND = no death within the follow-up period; MST = mean survival time in days.

**Table 2:** Effect of the hydromethanolic leaf extract of *M. dianthera* on rectal temperature and body weight of *P. berghei*-infected mice.

Test sample	Dose (mg/kg)	Rectal temperature (°C)			Body wt. (g)		
		*D*_0_	*D*_4_	% of change	*D*_0_	*D*_4_	% of change
NC	10 ml/kg	36.0 ± 0.40	35.05 ± 0.08	−2.58 ± 1.00	29.15 ± 0.70	28.70 ± 0.82	−1.53 ± 0.71
Extract	200	35.90 ± 0.24	35.48 ± 0.26	−2.23 ± 1.09	29.97 ± 0.70	31.67 ± 0.65	1.95 ± 1.32
Extract	400	36.02 ± 0.29	35.78 ± 0.45	−0.64 ± 0.90	32.48 ± 0.80	33.38 ± 0.55	5.89 ± 1.66
Extract	600	36.40 ± 0.18	35.58 ± 0.25	−1.14 ± 0.83	29.54 ± 2.11	30.66 ± 2.06	4.02 ± 1.50
CQ	25	35.90 ± 0.24	36.28 ± 0.13	1.07 ± 0.49^*∗*^	25.23 ± 0.56	25.54 ± 0.64	1.46 ± 2.82

Values are presented as Mean ± SEM, *n* = 6; CQ = chloroquine phosphate; NC = negative control; ^*∗*^*P* < 0.05 as compared to NC; *D*_4_ = day 4; *D*_0_ = day 0.

**Table 3:** Effect of solvent fractions of *M. dianthera* on mean parasitaemia level and mean survival time of *P. berghei*-infected mice.

Fraction/drug	Dose (mg/kg)	% of parasitaemia	% of suppression	MST (days)
NC	10 ml/kg	43.5 ± 1.52	0.00	7.33 ± 0.21
AF	200	36.27 ± 5.26	13.50	8 ± 0.45
	400	28.68 ± 3.00^a^^*∗*^	34.07	8.5 ± 0.5
BF	200	38.07 ± 1.92	13.30	8.17 ± 0.60
	400	31.90 ± 3.42	23.30	7.67 ± 0.21^b^^*∗*^
CF	200	31.6 ± 1.56	24.30	8.5 ± 0.56
	400	28.18 ± 3.78^a^^*∗*^	35.21	10 ± 0.77^a^^*∗*^
EF	200	38.67 ± 1.42	7.51	8.17 ± 0.40
	400	34.70 ± 5.07	17.1	8 ± 0.37
CQ	25	0.00^a^^*∗∗*^	100.00	ND

Values are expressed as Mean ± SEM; *n* = 6; CQ = chloroquine phosphate; NC = negative control; AF = aqueous fraction; BF, butanol fraction; CF = chloroform fraction; EF = ethyl acetate fraction; ^*∗*^*P* < 0.05; ^*∗∗*^*P* < 0.001; ^a^when compared to NC; ^b^when compared to CF400 mg/kg; ND = no death within the follow-up period; MST = mean survival time in days.

**Table 4:** Effect of solvent fractions of *M. dianthera* on rectal temperature and body weight of *P. berghei*-infected mice.

Test sample	Dose (mg/kg)	Rectal temperature (°C)			Body wt. (g)		
		*D*_0_	*D*_4_	% of change	*D*_0_	*D*_4_	% of change
NC	10 ml/kg	36.35 ± 0.16	35.5 ± 0.32	−2.32 ± 1.17	25.48 ± 2.22	25.07 ± 2.07	−1.40 ± 1.40
AF	200	36.1 ± 0.36	35.53 ± 0.31	−1.67 ± 1.11^b^^*∗*^	24.3 ± 1.75	25.9 ± 2.03	6.38 ± 1.24^d^^*∗∗*^^e^^*∗*^
	400	35.9 ± 0.23	36.33 ± 0.25	1.22 ± 0.80^c^^*∗∗∗*^	24.3 ± 1.75	25.9 ± 2.03	6.38 ± 1.24^d^^*∗∗*^^e^^*∗*^
BF	200	36.8 ± 0.46	36.97 ± 0.29	0.47 ± 1.28^c^^*∗∗∗*^	24.23 ± 1.79	26.25 ± 0.81	8.5 ± 1.3^c^^*∗*^^e^^*∗*^^d^^*∗∗∗*^
	400	36.8 ± 0.41	36.17 ± 0.19	−1.79 ± 1.1^b^^*∗∗∗*^	24.02 ± 1.65	25.82 ± 0.94	8.5 ± 3.1^c^^*∗*^^d^^*∗∗∗*^^e^^*∗*^
CF	200	36 ± 0.25	36.1 ± 0.19	0.29 ± 0.69^c^^*∗∗∗*^	28.43 ± 0.84	29.38 ± 0.71	3.43 ± 0.84^d^^*∗*^
	400	35.17 ± 1.24	36.17 ± 1.04	2.98 ± 0.84^d^^*∗∗*^	26.83 ± 2.48	27.68 ± 0.11	3.87 ± 1.85^d^^*∗*^
EF	200	36.68 ± 0.17	34.13 ± 0.21	−6.9 ± 0.9^a^^*∗∗∗*^^e^^*∗*^	22.13 ± 0.80	23.65 ± 0.97	5.23 ± 2.19^d^^*∗*^^e^^*∗*^
	400	36.03 ± 0.11	35.52 ± 0.19	−0.43 ± 0.61^bc^^*∗*^	23.18 ± 1.13	25.37 ± 1.12	9.6 ± 1.8^c^^*∗*^^d^^*∗∗∗*^^e^^*∗*^
CQ	25	36.87 ± 0.30	36.92 ± 0.29	0.15 ± 0.47^c^^*∗∗*^	30.67 ± 1.14	30.9 ± 0.73	1.04 ± 1.67

Values are presented as Mean ± SEM; *n* = 6; CQ, chloroquine phosphate; NC, negative control; AF, aqueous fraction; BF, butanol fraction; CF, chloroform fraction; EF, ethyl acetate fraction; ^*∗*^*P* < 0.05; ^*∗∗*^*P* < 0.01; ^*∗∗∗*^*P* < 0.001; ^a^compared to AF 400 mg/kg; ^b^compared to CF 400 mg/kg; ^c^compared to EF 200 mg/kg; ^d^compared to NC; ^e^compared to CQ treated group.

## Data Availability

The data sets used and/or analyzed during the current study are available from the corresponding author on reasonable request.
